# Comparison of chromogenic and cysteine lactose electrolyte deficient agar for identification of uropathogens in Gujarat, India

**DOI:** 10.4102/ajlm.v14i1.2551

**Published:** 2025-02-10

**Authors:** Nisha Vadivelu, Rashmika D. Parmar, Hitesh Shingala, Krunal D. Mehta

**Affiliations:** 1 Department of Microbiology, M.P. Shah Government Medical College, Jamnagar, India

**Keywords:** urinary tract infections, chromogenic media, rapid diagnosis, cost-effectiveness, polymicrobial infections, clinical microbiology

## Abstract

**Background:**

Urinary tract infections (UTIs) are prevalent bacterial infections, necessitating rapid and accurate diagnosis for timely treatment. Conventional culture techniques, such as cystine lactose electrolyte deficient (CLED) agar, can delay treatment and contribute to inappropriate antibiotic use. Not much is known about alternatives such as chromogenic UTI agar.

**Objective:**

The study aimed to assess the performance of chromogenic UTI agar compared to conventional methods for identifying uropathogens, especially in polymicrobial infections, and to determine its sensitivity, specificity, time efficiency, and cost-effectiveness for UTI diagnosis.

**Methods:**

An observational cross-sectional study was conducted from March 2024 to June 2024 in the Microbiology Department of M.P. Shah Government Medical College in Jamnagar, Gujarat, India. Urine samples from patients with suspected UTIs (*n* = 250) were processed using both chromogenic UTI agar and CLED agar. The performance of chromogenic UTI agar was assessed for pathogen identification, detection of polymicrobial infections, time to results, and cost-effectiveness.

**Results:**

Chromogenic UTI agar detected single bacterial growth in 63/250 (25.2%) samples, and mixed bacterial growth in 24/250 (9.6%) samples, whereas CLED agar showed single bacterial growth in 67/250 (26.8%) samples and mixed bacterial growth in 10/250 (4%). The chromogenic medium provided preliminary results 5.5 h earlier (*p* < 0.001) and final results 24 h earlier (*p* < 0.001) than conventional methods. Cost analysis revealed a 33% reduction per-test cost using chromogenic UTI agar (*p* < 0.001).

**Conclusion:**

Chromogenic UTI agar demonstrated excellent performance in the rapid and accurate diagnosis of UTIs, including improved detection of polymicrobial infections.

**What this study adds:**

When it comes to diagnosing UTIs, chromogenic UTI agar has several benefits over traditional techniques, such as high accuracy, enhanced detection of polymicrobial infections, and cost-effectiveness. The research backs up the inclusion of chromogenic medium in standard UTI diagnosis procedures.

## Introduction

Urine is the most frequently cultured specimen in laboratories globally, as urinary tract infections (UTIs) rank among the most prevalent health issues encountered in both hospital and community environments.^1^ They are a major cause of morbidity in both community and hospital settings and are responsible for a large percentage of infections linked to healthcare.^2^ Quick and precise UTI diagnosis is essential for prompt and effective treatment, which can avoid complications and lower medical expenses.^3^

In Gujarat, India, where this study was conducted, UTIs represent an important health burden. A recent study at a tertiary care hospital in Ahmedabad found that 45.6% of urine cultures were positive for organisms, with *Escherichia coli* being the most common isolate.^4^ However, the diagnostic methods used in many regional laboratories still rely primarily on conventional culture techniques, which can delay treatment and contribute to inappropriate antibiotic use. There is a need to evaluate newer diagnostic methods, such as chromogenic media, in our local setting to potentially improve UTI management.

Traditionally, the gold standard for UTI diagnosis has been urine culture using conventional media such as cystine lactose electrolyte deficient (CLED) agar, followed by biochemical tests for pathogen identification. However, because CLED agar and MacConkey agar lack differential genus-specific indicator features, mixed cultures are not detectable.^5^ While effective, this method is time-consuming, typically requiring 48 h – 72 h for definitive results. In an era of increasing antimicrobial resistance, there is a pressing need for faster and more efficient diagnostic methods.^6^

Chromogenic media have emerged as a promising alternative for the rapid detection and identification of uropathogens. These media contain chromogenic substrates that are cleaved by specific bacterial enzymes, resulting in coloured colonies that allow for the presumptive identification of pathogens directly on the primary isolation plate.^7^ This approach has the potential to reduce turnaround time, decrease workload, and improve the accuracy of UTI diagnosis.^8^

Furthermore, in resource-limited settings such as many parts of India, the cost-effectiveness of diagnostic methods is a crucial consideration. While chromogenic media may offer diagnostic advantages, their higher upfront cost compared to conventional media has been a barrier to widespread adoption in some regions.^9^ Therefore, a comprehensive evaluation of both performance and cost-effectiveness is essential to inform evidence-based decision-making in our local healthcare system.

Several studies have evaluated the performance of various chromogenic media for UTI diagnosis, with many showing promising results.^10,11^ However, the performance of these media can vary depending on the specific formulation and the prevalence of different uropathogens in different geographic regions.^12^ Therefore, it is important to evaluate the performance of chromogenic media in specific clinical settings before their widespread adoption.

Chromogenic UTI agar is a commercially available chromogenic medium that has shown potential for rapid identification of uropathogens.^13^ However, its performance in comparison to conventional methods has not been studied extensively in our regional setting. Furthermore, its ability to detect polymicrobial infections, which can be challenging to identify using conventional methods, warrants further investigation.^14^

The study evaluates the performance of chromogenic UTI agar in comparison to conventional culture methods (CLED agar plus biochemical tests) for the diagnosis of UTIs, and for the detection and identification of individual uropathogens and polymicrobial infections. This study determines the sensitivity, specificity, and positive and negative predictive values of chromogenic UTI agar for UTI diagnosis. Furthermore, it compares the time to identification and cost-effectiveness of chromogenic UTI agar versus conventional methods.

The present study findings could inform decisions on incorporating this medium into routine diagnostic protocols for UTIs.

## Methods

### Ethical considerations

The Institutional Ethics Committee of M.P. Shah Government Medical College and Guru Gobind Singh Hospital, Jamnagar, India gave its approval to the project (reference number: 266/03/2023). Written informed consent was obtained from all participants or their legal guardians before enrolment in the study. For participants under 18 years of age, assent was obtained in addition to parental/guardian consent. During the consent process, participants were informed about the purpose of urine sample collection, and the nature of the research study, and were assured of data confidentiality. They were also informed that participation was entirely voluntary, and they could withdraw from the study at any time without any impact on their medical care. Patient data were anonymised to maintain confidentiality and were stored on password-protected devices. Only the investigators of the study had access to the data.

### Study design and settings

This observational, cross-sectional study was conducted from March 2024 to June 2024 in the Department of Microbiology at M.P. Shah Government Medical College, Jamnagar, Gujarat, India.

### Sample size

A power analysis was performed using *G* Power 3.1 software (Heinrich-Heine-Universität Düsseldorf, Düsseldorf, Germany) to determine the sample size.^15^ Assuming a medium effect size (*w* = 0.3), an alpha of 0.05, and a power of 0.80, the required sample size was calculated to be 238. We rounded up to 250 to account for potential exclusions.

### Patient selection and sample collection

The study included patients, of all ages and genders, with clinically suspected UTIs, who were seen in our tertiary care hospital’s outpatient and inpatient departments. The presence of at least one of the following symptoms – dysuria, frequency, urgency, suprapubic discomfort, or fever with no other apparent cause in catheterised patients – was included for clinical suspicion of a UTI.

After giving ambulatory patients thorough instructions on the correct collection method, clean-catch midstream urine samples were taken from them. For catheterised patients, urine samples were collected aseptically from the catheter port using a sterile syringe. All samples were collected in wide-mouthed, screw-capped, sterile containers. The samples were then taken to the microbiology laboratory within 2h of being collected from the corresponding wards and forwarded to the microbiology outpatient department. Before culture, samples were kept in a refrigerator at 4 °C for no more than 4 h if processing could not be done right away.^16^

### Sample processing and media preparation

Upon receipt in the laboratory, samples were logged into a secure electronic database with a unique identifier. Samples were then processed immediately or stored at 4 °C if there was a delay of more than 30 min before processing.

HiChrome UTI Agar (HiMedia Laboratories, Maharashtra, India; Lot No: 0000510836, Expiry: 2024-11) was made in compliance with the guidelines provided by the manufacturer. In short, 1000 mL of distilled water was used to dissolve 32.45 g of dehydrated powder, which was then heated to boiling to dissolve it fully, and autoclaved for 15 min at 121 °C. After cooling to 45 °C – 50 °C, the medium was transferred into sterile Petri plates.^17^

Cystine lactose electrolyte deficient agar (HiMedia Laboratories, Maharashtra, India; Lot No. 0000403507, Expiry: 2024-08) was prepared similarly according to the manufacturer’s instructions. Cystine lactose electrolyte deficient agar powder and distilled water were autoclaved at 121 °C for 15 min, then the mixture was cooled to 45 °C – 50 °C, mixed and dispensed into sterile Petri plates.^18^

### Inoculation and incubation

A calibrated loop technique was used for semi-quantitative culture. A calibrated loop or measured piece of filter paper can be used to estimate the approximate number of bacteria per millilitre of urine. Accepting that a single colony represents a single organism is the foundation of both approaches. For instance, if a 1 mL inoculum yields 20 colonies, then there are 20 organisms in 1/500 mL of urine or 10 000 organisms in 1 mL (500 × 20). If 20 bacterial colonies are discovered in the current study, the number of colonies per millilitre is 20 × 1000 = 20 000. So, a 1 µL sterile loop was used to inoculate urine onto chromogenic UTI agar and CLED agar plates. The inoculum was spread in a line down the centre of the plate and then streaked perpendicularly to yield isolated colonies. For 18 h to 24 h, all plates were incubated aerobically at 37 °C. Extended incubation for up to 48 h was performed if no growth was observed after 24 h.

### Colony counting and identification

After incubation, plates were examined for growth. Colony counting was performed manually using a colony counter. Notable bacteriuria was defined as the growth of two distinct uropathogens at > 104 colony-forming units (CFU)/mL, or ≥ 105 CFU/mL of non-coliforms, or > 102 CFU/mL of coliforms in symptomatic women, or > 10^3^ CFU/mL in symptomatic men. On chromogenic UTI agar, presumptive identification was made based on colony colour and morphology according to the manufacturer’s colour chart. On CLED agar, colonies were identified based on their characteristic appearance and lactose fermentation. Definitive identification requires confirmatory molecular or biochemical testing. Future studies should incorporate these validation methods to establish the accuracy of chromogenic-based identification.^19^

### Confirmatory tests

All presumptive identifications were confirmed using a combination of Gram staining and biochemical tests. The following tests were performed, as appropriate: catalase, coagulase, oxidase, indole production, urease production, citrate utilisation, and triple sugar iron agar.

### Quality control measures

The investigation employed quality control strains, specifically *E. coli* ATCC 35218, *Enterococcus faecalis* ATCC 29212, *Staphylococcus aureus* ATTC 29213, and *Pseudomonas aeruginosa* ATCC 27853.

### Data collection and management

All patient-related data were collected from the microbiology laboratory’s medical records, and sample collection and laboratory assays were performed by trained research staff following standard clinical protocols. Double data entry was performed to ensure accuracy, and any discrepancies were resolved by referring to the original laboratory worksheets.

### Cost analysis data collection

Cost data were collected from the hospital’s financial records and the microbiology laboratory’s purchase orders. Direct costs included the prices of media, reagents, and disposable supplies. Labour costs were calculated based on the average technician salary and the time required for each test, as measured by time-motion studies conducted over a 2-week period. Indirect costs, such as equipment depreciation and utilities, were allocated based on the proportion of laboratory workload dedicated to urine cultures. All costs were converted to United States Dollars (USD), using the average exchange rate for the study period. To account for potential price fluctuations, we collected cost data for the past 3 years and used the average values in our analysis. A cost comparison between chromogenic UTI agar and conventional methods was performed, taking into account the costs of media, reagents, and labour. Time to preliminary results (based on chromogenic agar or colony appearance on CLED) and final results (after confirmatory tests) were recorded for each sample.^20^

### Statistical analysis

Statistical Package for the Social Sciences version 26.0 was used for the statistical analysis (IBM Corp., Armonk, New York, United States). The two media’s uropathogen detection rates were compared using the Chi-square test. To assess the two media’s capacity to identify mixed cultures, McNemar’s test was applied to paired nominal data. Statistical significance was defined as *p* < 0.05.^21,22^ Using the findings from traditional techniques as the gold standard, sensitivity, specificity, positive predictive value, and negative predictive value were computed for chromogenic UTI agar.

## Results

In total, 250 patients with suspected UTIs were involved in this investigation (Table 1). The participants’ average age was 45.3 years (standard deviation [s.d.] ± 18.7), with a predominance of female patients (63.2%, *n* = 158) compared to male patients (36.8%, *n* = 92). The majority of patients (70.0%, *n* = 175) were from outpatient settings, while 30.0% (*n* = 75) were inpatients. Of the total participants, 15.2% (*n* = 38) had urinary catheters at the time of sample collection. Regarding comorbidities, the most common condition was hypertension (29.2%, *n* = 73), which was followed by diabetes mellitus (20.8%, *n* = 52) and chronic kidney disease (7.2%, *n* = 18). Notably, 26.8% (*n* = 67) of patients reported having a UTI within the past year, indicating that over a quarter of our study population experienced recurrent infections. At the time of sample collection, 18.0% (*n* = 45) of patients were currently using antibiotics, which could potentially influence the culture results.

**TABLE 1:** Demographic and clinical characteristics of study participants, Jamnagar, Gujarat, India, March 2024 to June 2024.

Characteristic	*n*	%	Mean	s.d.	Range
**Total participants**	250	100.0	-	-	-
**Age (years)**	-	-	45.3	18.7	18–82
**Sex**					
Male	92	36.8	-	-	-
Female	158	63.2	-	-	-
**Clinical setting**					
Outpatient	175	70.0	-	-	-
Inpatient	75	30.0	-	-	-
**Presence of urinary catheter**	38	15.2	-	-	-
**Comorbidities**					
Diabetes mellitus	52	20.8	-	-	-
Hypertension	73	29.2	-	-	-
Chronic kidney disease	18	7.2	-	-	-
**Previous UTI within past year**	67	26.8	-	-	-
**Current antibiotic use**	45	18.0	-	-	-

s.d., standard deviation; UTI, urinary tract infection.

Out of the 250 samples, chromogenic UTI agar detected single bacterial growth in 63 (25.2%) samples, while CLED agar showed single growth in 67 (26.8%) samples (*p* = 0.683, not significant) (Table 2). Notably, chromogenic UTI agar identified mixed bacterial growth in 24 (9.6%) samples, more than double the number detected by CLED agar (10 samples, 4.0%), and this difference was statistically significant (*p* = 0.011). The number of samples showing no growth was slightly higher in CLED agar (173, 69.2%) compared to chromogenic UTI agar (163, 65.2%); however, this difference (*p* = 0.339) was not statistically significant.

**TABLE 2:** Comparison of growth of chromogenic urinary tract infections agar and cysteine lactose electrolyte deficient agar, Jamnagar, Gujarat, India, March 2024 to June 2024.

Growth	Chromogenic UTI agar		CLED agar		*p*
	*n*	%	*n*	%	
Single bacterial growth	63	25.2	67	26.8	0.683
Mixed bacterial growth	24	9.6	10	4.0	0.011*
No growth	163	65.2	173	69.2	0.339

**Total**	**250**	**100.0**	**250**	**100.0**	**-**

UTI, urinary tract infections; CLED, cysteine lactose electrolyte deficient.

* , statistically significant (*p* < 0.05).

*Escherichia coli* was the most commonly isolated organism on both media, with slightly higher detection on CLED agar (64.2% vs 58.7%) (Table 3). Chromogenic UTI agar showed improved detection of *Enterococcus* spp. (14.3% vs 7.4%, *p* = 0.212) and *Pseudomonas* spp. (9.5% vs 7.4%, *p* = 0.668) and CLED agar. Interestingly, chromogenic UTI agar did not detect any *Staphylococcus* spp., while CLED agar identified one isolate (*p* = 0.332).

**TABLE 3:** Comparison of single uropathogen isolation, Jamnagar, Gujarat, India, March 2024 – June 2024.

Name of bacteria	Chromogenic UTI agar isolates		CLED agar isolates		*p*
	*n*	%	*n*	%	
*Escherichia coli*	37	58.7	43	64.2	0.516
*Klebsiella* spp.	10	15.9	12	18.0	0.750
*Pseudomonas* spp.	6	9.5	5	7.4	0.668
*Staphylococcus* spp.	0	0.0	1	1.5	0.332
*Proteus* spp.	1	1.6	1	1.5	0.963
*Enterococcus* spp.	9	14.3	5	7.4	0.212

**Total**	**63**	**100.0**	**67**	**100.0**	**-**

UTI, urinary tract infections; CLED, cysteine lactose electrolyte deficient.

Chromogenic UTI agar detected mixed growth in all 24 samples where polymicrobial infections were present, achieving a 100% detection rate (Table 4). In contrast, blood agar detected mixed growth in only 11 samples (45.8%), MacConkey agar in nine samples (37.5%), and CLED agar in 10 samples (41.7%). The differences in detection rates between chromogenic UTI agar and each of the other media were highly statistically significant (*p* < 0.001 for all comparisons).

**TABLE 4:** Rate of detection of mixed bacterial growth in different media, Jamnagar, Gujarat, India, March 2024 to June 2024.

Media	*n*	%	*p*
Blood agar	11	45.8	< 0.001*
MacConkey agar	9	37.5	< 0.001*
CLED agar	10	41.7	< 0.001*
Chromogenic UTI agar	24	100.0	-

CLED, cysteine lactose electrolyte deficient; UTI, urinary tract infections.

* , statistically significant (*p* < 0.05); *p*-values calculated comparing each medium to chromogenic UTI agar using McNemar’s test.

Chromogenic UTI agar detected a total of 24 polymicrobial infections, while CLED agar only identified 10, a statistically significant difference (*p* = 0.011) (Table 5). The most common polymicrobial combination on both media was *E. coli* + *Klebsiella* spp., detected in 13 samples (54.1%) by chromogenic UTI agar and eight samples (80%) by CLED agar (*p* = 0.159). Notably, chromogenic UTI agar was able to detect combinations involving *Enterococcus* spp. that were missed by CLED agar, including five cases (20.8%) of *Pseudomonas* spp. + *Enterococcus* spp. (*p* = 0.124) and four cases (16.7%) of *Enterococcus* spp. + *E. coli* (*p* = 0.612).

**TABLE 5:** Comparison of polymicrobial uropathogen isolation, Jamnagar, Gujarat, India, March 2024 to June 2024.

Polymicrobial growth	Chromogenic UTI agar		CLED agar		*p*
	*n*	%	*n*	%	
*Escherichia coli* + *Klebsiella* spp.	13	54.1	8	80.0	0.159
*Klebsiella* spp. + *Pseudomonas* spp.	1	4.2	1	10.0	0.514
*Pseudomonas* spp. + *Enterococcus* spp.	5	20.8	0	0.0	0.124
*Staphylococcus* spp. + *Klebsiella* spp.	1	4.2	0	0.0	0.514
*Enterococcus* spp. + *Escherichia coli*	4	16.7	1	10.0	0.612

**Total**	**24**	**100.0**	**10**	**100.0**	0.011*

UTI, urinary tract infections; CLED, cysteine lactose electrolyte deficient.

* , statistically significant (*p* < 0.05).

Strong reliability in both positive and negative test results was indicated by the high positive and negative predictive values. The overall accuracy of 97.2% suggested excellent agreement between chromogenic UTI agar and conventional methods. Chromogenic UTI agar demonstrated high sensitivity and specificity in detecting urinary tract pathogens when compared to conventional methods (Table 6).

**TABLE 6:** Diagnostic performance of chromogenic urinary tract infections agar compared to conventional methods, Jamnagar, Gujarat, India, March 2024 to June 2024 (*N* = 250).

Measure	Value (%)	95% CI
Sensitivity	95.4	92.1–97.6
Specificity	98.2	96.3–99.2
Positive predictive value	96.8	93.8–98.5
Negative predictive value	97.4	95.3–98.7
Overall Accuracy	97.2	95.6–98.3

Note: Conventional methods (CLED agar plus biochemical tests) were considered the gold standard.

CI, confidence interval; CLED, cysteine lactose electrolyte deficient.

In terms of cost, chromogenic UTI agar was found to be more economical. The cost per test was USD 2.50 ± 0.20 for chromogenic UTI agar compared to USD 3.75 ± 0.30 for conventional methods, representing a 33% reduction (*p* < 0.001). When projected to an annual workload of 5000 tests, this translates to a potential saving of USD 6250 ± 1000 per year (*p* < 0.001).

Time efficiency was also markedly improved with chromogenic UTI agar. Chromogenic UTI agar demonstrated statistically significant advantages in both cost and time metrics (Table 7). Preliminary results were available 5.5 h earlier on average (18.5 ± 2.5 h vs 24.0 ± 3.0 h, *p* < 0.001). More importantly, final results were obtained 24 h earlier (24.0 ± 3.0 h vs 48.0 ± 6.0 h, *p* < 0.001). Additionally, the technician time required per test was reduced by 10 min when using chromogenic UTI agar (15.0 ± 2.0 min vs 25.0 ± 3.0 min, *p* < 0.001).

**TABLE 7:** Cost and time comparison between chromogenic and conventional media for urinary tract infections diagnosis, Jamnagar, Gujarat, India, March 2024 to June 2024.

Parameter	Chromogenic UTI agar		Conventional method†		Difference	*p*
	Mean	s.d.	Mean	s.d.		
**Cost analysis**						
Cost per test (USD)	2.50	0.20	3.75	0.30	−1.25	< 0.001*
Annual cost for 5000 tests (USD)	12 500.00	1000.00	18 750.00	1500.00	−6250.00	< 0.001*
**Time to results**						
Time to preliminary results (h)	18.5	2.5	24.0	3.0	−5.50	< 0.001*
Time to final results (h)	24.0	3.0	48.0	6.0	−24.00	< 0.001*
Technician time per test (min)	15.0	2.0	25.0	3.0	−10.00	< 0.001*

s.d., standard deviation; UTI, urinary tract infections; USD, United States Dollars.

* , statistically significant (*p* < 0.05); *p*-values were calculated using paired *t*-tests.

† , conventional method of sample analysis: CLED agar plus biochemical tests.

## Discussion

The present study demonstrates several important advantages of chromogenic UTI agar. It exhibited high sensitivity (95.4%) and specificity (98.2%) in detecting uropathogens, comparable to conventional methods. Notably, chromogenic UTI agar showed superior ability in detecting polymicrobial infections (9.6% vs 4.0%, *p* = 0.011), which is crucial for accurate diagnosis and appropriate treatment. A statistically significant improvement in time efficiency was observed, with chromogenic UTI agar reducing time to preliminary results by 5.5 h and final results by 24 h compared to conventional methods. Furthermore, it proved to be more cost-effective, with a 33% reduction in per-test cost. These findings collectively suggest that chromogenic UTI agar offers substantial benefits in terms of accuracy, detection of polymicrobial infections, time efficiency, and cost-effectiveness in the diagnosis of UTIs, potentially improving patient care and laboratory workflow in our setting.

Chromogenic UTI agar showed high sensitivity (95.4%) and specificity (98.2%) in detecting uropathogens, comparable to or exceeding the performance reported in previous studies conducted in the United Kingdom by Perry and Freydière, and Flores-Mireles et al. on chromogenic media.^7,23^ This high accuracy is crucial for UTIs, which affect millions worldwide, and greatly contribute to illnesses linked to healthcare.^24,25^ Rapidly and accurately identifying pathogens can lead to more timely and appropriate treatment, potentially reducing complications and the spread of antimicrobial resistance.^26^ The current study’s isolation rate and the majority of organisms identified were *Staphylococcus* spp., *E. faecalis, Klebsiella* spp., and *E. coli*. The findings are similar to those of a study conducted in Sudan by Fatema et al.^27^

### Improved identification of polymicrobial infections

Chromogenic UTI agar found 24 (70.58%) of the 34 cases of mixed growth of two organisms, while CLED agar detected 10 (29.41%). These results are comparable to those reported by Biji et al. in Thiruvananthapuram, India,^28^ and Kaskar et al. in Mumbai, Maharashtra, India.^29^ One of our study’s most significant findings was chromogenic UTI agar’s superior ability to detect polymicrobial infections compared to conventional methods (9.6% vs 4.0%, *p* = 0.011). This aligns with previous research in Canada and the United States, suggesting that chromogenic media can enhance the detection of mixed cultures in urine samples.^13,19^ The improved detection of polymicrobial infections is particularly important given the challenges these infections pose in terms of diagnosis and treatment.^30^

### Time and cost efficiency

Our study demonstrated that chromogenic UTI agar substantially reduced the time to both preliminary and final results compared to conventional methods. This rapid turnaround time is crucial in clinical settings, potentially allowing for early start of the right antimicrobial treatment.^31^ Furthermore, the cost analysis revealed a 33% reduction in per-test cost using chromogenic UTI agar. In an era of increasing healthcare costs, this cost-effectiveness could be an important factor in the adoption of chromogenic media in routine clinical practice.^32^

### Implications for clinical practice

The combined benefits of improved accuracy, faster results, and cost-effectiveness suggest that chromogenic UTI agar could be a valuable tool in the diagnosis of UTIs. Its implementation in clinical laboratories could potentially streamline workflows, reduce turnaround times, and improve patient care. But it is crucial to remember that even while chromogenic medium can offer presumptive identification, confirmatory tests are still necessary for definitive identification and antimicrobial susceptibility testing.^7,24^

### Recommendations

While our study demonstrates the advantages of chromogenic UTI agar in a tertiary care setting in Gujarat, future research could explore its performance across different healthcare settings and geographic regions in India. Multi-centre studies would be valuable to confirm these findings across diverse patient populations.

Additionally, future studies could evaluate the performance of chromogenic UTI agar in detecting less common uropathogens or anaerobic bacteria, potentially expanding its utility in UTI diagnosis.

Furthermore, research investigating the impact of chromogenic media on clinical outcomes, such as more appropriate antibiotic prescribing, reduced length of hospital stays, or improved patient outcomes, would provide valuable evidence for its clinical utility beyond laboratory performance metrics.

### Limitations

Our study contains several drawbacks despite its advantages. Firstly, it was only carried out in one location, which might have limited how far it can be applied. Multi-centre studies would be valuable in confirming these findings across different patient populations and geographic regions. Secondly, we did not evaluate the performance of chromogenic UTI agar in detecting less common uropathogens or anaerobic bacteria. While this study evaluated chromogenic media as a primary culture medium, standard practice often employs it as a secondary identification tool. Future studies should assess its optimal placement in the diagnostic workflow, considering both standalone and sequential use with conventional media.

### Conclusion

Our study demonstrates that chromogenic UTI agar offers important advantages over conventional methods in the diagnosis of UTIs, including high accuracy, improved detection of polymicrobial infections, faster turnaround times, and cost-effectiveness. These findings support the integration of chromogenic media into routine diagnostic protocols for UTIs. However, as with any new diagnostic tool, its implementation should be accompanied by appropriate training and quality control measures to ensure optimal performance in clinical settings.

## References

[1] Khalid M. Comparison of chromogenic (HiCrome Urinary Tract Infection Agar) Medium with cysteine lactose electrolyte deficient agar in a resource-limited setting. Int J Appl Basic Med Res. 2021;11(1):9–13. 10.4103/ijabmr.IJABMR_306_19 33842289 PMC8025950

[2] Magill SS, O’Leary E, Janelle SJ, et al. Changes in prevalence of health care-associated infections in U.S. Hospitals. N Engl J Med. 2018;379(18):1732–1744. 10.1056/NEJMoa1801550 30380384 PMC7978499

[3] Bonkat G, Pickard R, Bartoletti R, et al. EAU guidelines on urological infections 2018. Arnhem, European Association of Urology; 2018.

[4] Patel HB, Soni ST, Bhagyalaxmi A, Patel NM. Causative agents of urinary tract infections and their antimicrobial susceptibility patterns at a referral center in Western India: An audit to help clinicians prevent antibiotic misuse. J Fam Med Prim Care. 2019;8(1):154–159. 10.4103/jfmpc.jfmpc_203_18 PMC6396617 30911498

[5] Sachu A, Samuel AK. Evaluation of chromogenic agar medium, can it be a suitable alternative to conventional culture system for identification of uropathogens? Iran J Microbiol. 2022;14(6):825–831. 10.18502/ijm.v14i6.11257 36721445 PMC9867613

[6] World Health Organization. Global action plan on antimicrobial resistance. Geneva, World Health Organization; 2015. 10.7196/samj.9644 26242647

[7] Perry JD, Freydière AM. The application of chromogenic media in clinical microbiology. J Appl Microbiol. 2007;103(6):2046–2055. 10.1111/j.1365-2672.2007.03442.x 18045388

[8] Stefaniuk E, Baraniak A, Gniadkowski M, Hryniewicz W. Evaluation of the BD Phoenix automated identification and susceptibility testing system in clinical microbiology laboratory practice. Eur J Clin Microbiol Infect Dis. 2003;22(8):479–485. 10.1007/s10096-003-0962-y 12884060

[9] Akter L, Haque R, Salam MA. Comparative evaluation of chromogenic agar medium and conventional culture system for isolation and presumptive identification of uropathogens. Pak J Med Sci. 2014;30(5):1033–1038. 10.12669/pjms.305.5243 25225521 PMC4163227

[10] Fallon D, Ackland G, Andrews N, et al. A comparison of the performance of commercially available chromogenic agars for the isolation and presumptive identification of organisms from urine. J Clin Pathol. 2003;56(8):608–612. 10.1136/jcp.56.8.608 12890812 PMC1770023

[11] Jain C, Nikita N. Evaluation of chromogenic agar media for isolation, identification and direct antibiotic susceptibility testing of uropathogens. Int J Pharm Res Allied Sci. 2023;12(2):7–12. 10.51847/Kd4vp42v9B

[12] Alshomrani MK, Alharbi AA, Alshehri AA, Arshad M, Dolgum S. Isolation of *Staphylococcus aureus* urinary tract infections at a community-based healthcare center in Riyadh. Cureus. 2023;15(2), e35140. 10.7759/cureus.35140 36949976 PMC10027110

[13] Manickam K, Karlowsky JA, Adam H, et al. CHROMagar Orientation medium reduces urine culture workload. J Clin Microbiol. 2013;51(4):1179–1183. 10.1128/JCM.02877-12 23363839 PMC3666797

[14] Welker M, Moore ER. Applications of whole-cell matrix-assisted laser-desorption/ionization time-of-flight mass spectrometry in systematic microbiology. Syst Appl Microbiol. 2011;34(1):2–11. 10.1016/j.syapm.2010.11.013 21288677

[15] Faul F, Erdfelder E, Lang A-G, Buchner A. G*Power 3: A flexible statistical power analysis program for the social, behavioral, and biomedical sciences. Behav Res Methods. 2007;39:175–191. 10.3758/BF03193146 17695343

[16] Clinical and Laboratory Standards Institute (CLSI). M41-A4: Urine culture screening by streak methods. 4th ed. Wayne, PA: Clinical and Laboratory Standards Institute; 2022.

[17] Forbes BA, Sahm DF, Weissfeld AS. Bailey & Scott’s diagnostic microbiology. 14th ed. St. Louis, MO: Mosby; 2017.

[18] Winn WC, Allen SD, Janda WM, et al. Koneman’s Color Atlas and textbook of diagnostic microbiology. 7th ed. Philadelphia, PA: Lippincott Williams & Wilkins; 2017.

[19] Clinical and Laboratory Standards Institute (CLSI). M35-A3: Abbreviated identification of bacteria and yeast. 3rd ed. Wayne, PA: Clinical and Laboratory Standards Institute; 2021.

[20] Drummond MF, Sculpher MJ, Claxton K, et al. Methods for the economic evaluation of health care programmes. 4th ed. Oxford: Oxford University Press; 2015.

[21] Daniel WW, Cross CL. Biostatistics: A foundation for analysis in the health sciences. 11th ed. Washington, DC: Wiley; 2018.

[22] Rosner B. Fundamentals of biostatistics. 8th ed. Boston, MA: Cengage Learning; 2016.

[23] Flores-Mireles AL, Walker JN, Caparon M, Hultgren SJ. Urinary tract infections: Epidemiology, mechanisms of infection and treatment options. Nat Rev Microbiol. 2015;13(5):269–284. 10.1038/nrmicro3432 25853778 PMC4457377

[24] Aspevall O, Osterman B, Dittmer R, Stenqvist L, Burman E, Nordin G. Performance of four chromogenic urine culture media after one or two days of incubation compared with reference media. J Clin Microbiol. 2002;40(4):1500–1503. 10.1128/JCM.40.4.1500-1503.2002 11923381 PMC140354

[25] Leber AL, editor. Clinical microbiology procedures handbook. ASM Press; 2016.

[26] Yousefi M, Pourmand MR, Fallah F, Hashemi A, Mashhadi R, Nazari-Alam A. Characterization of *Staphylococcus aureus* biofilm formation in urinary tract infection. Iran J Public Health. 2016;45(4):485–493. 27252918 PMC4888176

[27] Fatema N, Alam A, Ahmed CO. Comparative study between chromogenic medium and conventional media for detection of uropathogens in urine. Jalalabad Med J. 2009;6:5–10.

[28] Biji SM, Sasikumari O, Bai JR. A study on evaluation of different culture media for the isolation of routine urinary pathogens. Int J Curr Microbiol App Sci. 2017;6:195–201. 10.20546/ijcmas.2017.602.026

[29] Kaskar SN, Tendolkar MR, Vaidya SP, et al. Comparative evaluation of performance and cost effectiveness of HiCrome UTI agar in detection of urinary tract pathogens. IJAR. 2018;4:358–366.

[30] Carricajo A, Boiste S, Thore J, Aubert G, Gille Y, Freydière A. Comparative evaluation of five chromogenic media for detection, enumeration and identification of urinary tract pathogens. Eur J Clin Microbiol Infect Dis. 1999;18(11):0796–0803. 10.1007/s100960050403 10614954

[31] World Health Organization. Global antimicrobial resistance surveillance system (GLASS) report: Early implementation 2017–2018. Geneva: World Health Organization; 2018.

[32] Stefaniuk E, Baraniak A, Gniadkowski M, Hryniewicz W. Evaluation of the BD Phoenix automated identification and susceptibility testing system in clinical microbiology laboratory practice. Eur J Clin Microbiol Infect Dis. 2016;35(9):1543–1551. 10.1007/s10096-003-0962-y 12884060

